# Adaptive and maladaptive perfectionism of graduating medical students

**DOI:** 10.1192/j.eurpsy.2022.1712

**Published:** 2022-09-01

**Authors:** L. Baranskaya, K. Zhuravskaya, V. Ivanova

**Affiliations:** Ural State Medical University, Psychiatry, Psychotherapy And Narcology, Yekaterinburg, Russian Federation

**Keywords:** perfectionism, graduating medical student

## Abstract

**Introduction:**

Perfectionism, as a multiform trait of character, plays an important role in the formation of motivation of achievements in socially significant activity. Adaptive perfectionism, together with the desire to achieve recognition in one’s professional community and insure the emotional stability. Maladaptive (neurotic) perfectionism is directed towards the achievement of excessively high (non-relevant) standards of activity, a constant anxiety, internal stress and lack of self-confidence

**Objectives:**

To pinpoint the types of perfectionism that graduating medical students at Medical University experience, those who, during all the years of study, showed high academic results

**Methods:**

Forty-nine graduating medical students volunteered to take part in the study, their average mark being not lower than 4.75 (maximum was 5). Their average age was 22.41±0.75. The following scales measured the level of expressiveness of perfectionism: A.A. Zolotareva, Hewitt and Flett, I.I. Gracheva

**Results:**

The results of the study undertaken showed two distinct groups. Students of the first group (79.6%) aimed at high internal standards in their study that would make them very well prepared professionally for their future work as doctors. They consciously accepted the common rules and norms of their society. Students of the other group (20.4%), consciously and subconsciously, estimate their high academic results as a good way of overcoming personal disturbances. They have excessive non-realistic demands towards self and others

**Conclusions:**

The results of our study make it possible for us to suppose that medical students of the second group will experience quite a lot of difficulties in their future professional activity

**Disclosure:**

No significant relationships.

